# A sugarcane mosaic virus vector for gene expression in maize

**DOI:** 10.1002/pld3.158

**Published:** 2019-08-08

**Authors:** Yu Mei, Guanjun Liu, Chunquan Zhang, John H. Hill, Steven A. Whitham

**Affiliations:** ^1^ Department of Plant Pathology and Microbiology Iowa State University Ames Iowa; ^2^ State Key Laboratory of Tree Genetics and Breeding Northeast Forestry University Harbin China; ^3^ Department of Agriculture Alcorn State University Lorman Mississippi

**Keywords:** gene expression, green fluorescent protein, maize, monocot, potyvirus, vector

## Abstract

*Zea mays* L. ssp. *mays* (maize) is an important crop plant as well as model system for genetics and plant biology. The ability to select among different virus‐based platforms for transient gene silencing or protein expression experiments is expected to facilitate studies of gene function in maize and complement experiments with stable transgenes. Here, we describe the development of a sugarcane mosaic virus (SCMV) vector for the purpose of protein expression in maize. An infectious SCMV cDNA clone was constructed, and heterologous genetic elements were placed between the protein 1 (P1) and helper component‐proteinase (HC‐Pro) cistrons in the SCMV genome. Recombinant SCMV clones engineered to express green fluorescent protein (GFP), β‐glucuronidase (GUS), or bialaphos resistance (BAR) protein were introduced into sweet corn (Golden × Bantam) plants. Documentation of developmental time courses spanning maize growth from seedling to tasseling showed that the SCMV genome tolerates insertion of foreign sequences of at least 1,809 nucleotides at the P1/HC‐Pro junction. Analysis of insert stability showed that the integrity of GFP and BAR coding sequences was maintained longer than that of the much larger GUS coding sequence. The SCMV isolate from which the expression vector is derived is able to infect several important maize inbred lines, suggesting that this SCMV vector has potential to be a valuable tool for gene functional analysis in a broad range of experimentally important maize genotypes.

## 1 INTRODUCTION

Virus‐based expression vectors are used to transiently and rapidly express a wide range of recombinant proteins in plants (Gleba, Klimyuk, & Marillonnet, 2007). The use of viruses to deliver heterologous proteins overcomes the need for transgenic plant production, which is time‐consuming and costly in most crop species. A variety of foreign proteins have been expressed from various viruses including reporter proteins (e.g., green fluorescent protein [GFP] and β‐glucuronidase [GUS]), selectable markers such as the bialaphos resistance (BAR) protein and biopharmaceutical proteins (e.g., vaccine epitopes and therapeutic proteins), and pathogen effectors (Bouton et al., 2018; Dawson & Folimonova, 2013; Gleba et al., 2007; Hefferon, 2012; Oh et al., 2009; Whitham, Yamamoto, & Carrington, 1999). The virus‐mediated expression of heterologous proteins is useful not only for *in planta* protein production, but also the use of reporter‐tagged viruses enables virus replication and movement to be easily tracked and quantified, which has greatly facilitated studies of virus–host interactions (Dolja, McBride, & Carrington, 1992). In addition, viruses expressing selectable markers enabled high throughput genetic screens for plant lines with altered virus susceptibility (Whitham et al., 1999).

Many viruses that infect dicot plants and belong to the *Potyvirus* genus have been engineered to express foreign proteins. An advantage of potyviruses is that their virions are filamentous, and therefore, the addition of a heterologous sequence results in a proportional increase in virion length (Kelloniemi, Makinen, & Valkonen, 2008). The mature viral proteins occur in the following order in the viral polyprotein: protein 1 (P1), helper component‐proteinase (HC‐Pro), protein 3 (P3), 6 kilo dalton 1 (6K1), cylindrical inclusion (CI), 6 kilo dalton 2 (6K2), viral protein genome‐linked (VPg), nuclear inclusion proteinase a (NIa‐Pro), nuclear inclusion b (NIb), and capsid protein (CP). The P1/HC‐Pro junction is cleaved in *cis* by the P1 proteinase, the HC‐Pro/P3 junction is cleaved in *cis* by HC‐Pro, and all other junctions are cleaved in *cis* or *trans* by NIa‐Pro. Potyviruses, including SCMV, encode an 11th protein, named PIPO, which is produced as a result of transcriptional slippage of the viral RNA polymerase at the amino‐terminus of the coding sequence of the P3 protein (Chung, Miller, Atkins, & Firth, 2008).

Because potyviruses use a polyprotein expression strategy, the coding sequences of foreign proteins must be cloned in‐frame with the viral open reading frame. In addition, the insertion site(s) for foreign sequences must be flanked by amino acids comprising viral proteinase cleavage sites to ensure that the recombinant protein is processed from the mature viral proteins. Six different locations have been shown to be suitable for expressing proteins from potyviral genomes (Chen et al., 2007; Fernandez‐Fernandez et al., 2001; Mavankal & Rhoads, 1991; Rajamaki et al., 2005; Varrelmann & Maiss, 2000; Verchot, Koonin, & Carrington, 1991). The two most commonly used cloning sites are at the junctions of P1/HC‐Pro and NIb/CP (Kelloniemi et al., 2008). P1 is a serine protease that cleaves its own C‐terminus from the N‐terminus of HC‐Pro (Mavankal & Rhoads, 1991; Verchot et al., 1991). Cloning sites using the P1/HC‐Pro junction are engineered immediately after the cleavage site, which results in cleavage of the P1 C‐terminus from the N‐terminus of the foreign protein. A seven amino acid NIa‐Pro cleavage site is added after the cloning site to process the C‐terminus of the foreign protein away from the N‐terminus of HC‐Pro (Carrington, Haldeman, Dolja, & Restrepo‐Hartwig, 1993). Similarly, cloning sites at the NIb/CP junction utilize the naturally occurring NIa‐Pro cleavage site at this junction along with an additional engineered NIa‐Pro cleavage site after the cloning site (Fernandez‐Fernandez et al., 2001; Varrelmann & Maiss, 2000).

Viruses in the sugarcane mosaic subgroup of the *Potyvirus* genus infect a wide range of plant species in the *Graminae*, including maize, sorghum, and sugarcane (Pirone, 1972). The sugarcane mosaic subgroup contains four closely related but distinct viral species: *Sugarcane mosaic virus* (SCMV), *Maize dwarf mosaic virus* (MDMV), *Johnson grass mosaic virus*, and *Sorghum mosaic virus* (Shukla et al., 1989). Similar to other potyviruses, SCMV has a positive sense, single‐stranded RNA genome that is polyadenylated at the 3′ terminus and encodes a large polyprotein that is cleaved into 10 mature proteins by three viral proteases (Chen, Chen, & Adams, 2002). Co‐infections of SCMV with the unrelated maize chlorotic mottle virus (MCMV) result in the destructive maize lethal necrosis disease that is a major problem for maize production in sub‐Saharan Africa (Redinbaugh & Stewart, 2018). The ability of SCMV to infect maize and other grass species where it may have utility for protein expression and its ability to participate in synergistic interactions with MCMV made SCMV an attractive candidate for developing infectious clones and expression vectors.

Here, we report the construction of an infectious cDNA clone derived from an isolate of SCMV that was originally identified as MDMV strain B (MDMV‐B) (Ford, Bucholtz, & Lambe, 1967). The viral genome was placed under control of the cauliflower mosaic virus 35S promoter (P35S) and the nopaline synthase terminator (Tnos), and the SCMV cDNA clone was modified to systemically express proteins from the P1/HC‐Pro junction in maize plants. The ability of SCMV to express foreign proteins was tested using green fluorescent protein (GFP), β‐glucuronidase (GUS), and bialaphos resistance (BAR) protein.

## 2 MATERIALS AND METHODS

### 2.1 Plants, virus strains, and inoculation

The SCMV virus isolate ([MDMV‐B] designated Iowa 66‐188 [ATCC‐PV53]) was first isolated in Iowa in 1966 (Ford et al., 1967; Hill, Ford, & Benner, 1973) and maintained in sweet corn (*Z. mays* cv. “Golden × Bantam”). Inoculum was prepared by grinding virus‐infected sweet corn leaves in 50 mM potassium phosphate buffer, pH 7.0 using a mortar and pestle. Leaves of 7‐ to 10‐day‐old (2‐leaf stage) sweet corn plants were dusted with 600‐mesh Carborundum (Buehler), and then they were mechanically inoculated by rubbing with a pestle dipped in the leaf sap. For biolistic inoculations, SCMV plasmids were introduced to leaves of 1‐week‐old plants using a Biolistic PDS‐1000/He system (Bio‐Rad Laboratories), as previously described for inoculation of maize seedlings with foxtail mosaic virus (FoMV) infectious clones (Mei & Whitham, 2018). Briefly, plants were placed in the dark 12 hr before bombardment. SCMV plasmid constructs were precipitated onto 1.0 μm gold particles, 1 μg of DNA coated onto gold particles was spread evenly on each macrocarrier, and leaves were bombarded using 1,100‐psi rupture disks at a distance of 6 cm. Bombarded plants were misted with water, covered with a clear plastic dome, and returned to the dark for 12 hr after bombardment. The plants were maintained in a greenhouse room or growth chamber at 20–22°C with a photoperiod of 16 hr.

### 2.2 Construction of infectious SCMV constructs

Plasmids produced for the initial SCMV constructs were propagated in ElectroMax DH5α‐E cells (Invitrogen) and purified using the QiaPrep Spin MiniPrep kit (Qiagen), and the polymerase chain reaction (PCR) was performed using Takara PrimeSTAR^™^ HS DNA Polymerase (TaKaRa Bio Inc) and oligonucleotide primers from Integrated DNA Technologies. Nucleotide sequencing was done using the Big Dye Terminator DNA Sequencing Kit (Applied Biosystems), and the ABI Prism 310 genetic analyzer at the Iowa State University DNA Facility. Sequence analysis was performed using the Vector NTI program (Invitrogen).

Total RNA was extracted from SCMV‐infected sweet corn leaves by the Trizol method (Invitrogen). First‐strand cDNA was synthesized using 0.5 μg of mRNA, 0.5 μg oligo(dT)_20_ primer, 1 μl 10 mM dNTP, and Superscript III reverse transcriptase (Invitrogen) to a final volume of 20 μl. A 2 μl aliquot of first‐strand cDNA reverse transcription product each was used as template in two 100 μl PCR reactions to amplify the 5′ and 3′ends of the SCMV genomic cDNA with primer pairs SC‐5end/SC‐2916R and SC‐3end/SC‐2916F, respectively (Table S1). The PCR conditions were as follows: (a) 1 min of denaturing at 98°C; (b) three cycles of denaturing at 98°C for 10 s, annealing at 4°C for 12 s, and extension at 68°C for 6.5 minutes; (c) 30 cycles of denaturing at 98°C for 10 s, annealing at 52°C for 12 s, and extension at 68°C for 7 min; and (d) final extension at 68°C for 10 min. The PCR products were gel extracted and used together as template in an overlapping PCR reaction with primer pairs SC‐5end and SC‐3end for the generation of full‐length genomic cDNA of SCMV. The SCMV full‐length PCR products were gel extracted, treated with T4 DNA kinase, and ligated into *Stu*I‐digested and dephosphorylated pSMV‐NVEC plasmid (Wang, Eggenberger, Hill, & Bogdanove, 2006) to generate the construct pSCMV‐IA. Clones were screened by PCR with primer pair SC‐9118F and Nos‐Rev for correct insertion orientation. Correct clones were further confirmed by sequencing with the 35‐Seq primer (Table S1), and the entire SCMV‐IA genomic insertion was sequenced with the primers listed in Table S1.

### 2.3 SCMV sequencing from plants co‐inoculated with SC129, SC159, and SC163

RNA was extracted from plants that had been co‐inoculated with SC129, SC159, and SC163, and RT‐PCR was performed using the primers listed in (Table S1). Primer pairs 157F and 745R were used for fragment 1, primer pairs 1487F and 2120R for fragment 2, primer pairs 3338F and 4955R for fragment 3, primer pairs 6015F and 7897R for fragment 4, and primer pairs 8232F and 9614R for fragment 5. The PCR fragments were cloned into the pGEM‐T easy vector (Promega, Madison, WI, USA), and multiple clones from each construct were sequenced. A total of 141 clones were sequenced in 237 sequencing reactions of which 216 reactions provided readable results. These sequences were analyzed using Bioedit software.

### 2.4 Construction of a single SCMV infectious clone

The SC129 and SC159 plasmids were used for construction of a full‐length SCMV infectious clone in three steps. First, SC129 and SC159 were digested with *Xba*I and *Bbv*CI. The resulting 1.7 kb fragment from SC159 and the 12.7 kb backbone of SC129 were gel purified and ligated to generate the construct SC129f1. Second, the SC129 and SC159 plasmids were digested with *Sal*I. The resulting 2.1 kb fragment from SC129 and the 12.3 kb backbone of SC159 were gel purified and ligated to generate the construct SC129f2. Third, the SC129f2 and SC129f1 plasmids were digested with *Kpn*I and *Bsu*36I. A 4.1 kb fragment from SC129f2 after KpnI and Bsu36I digestion was ligated to the 10.3 kb fragment from similarly digested SC129f1 to generate the construct SC129f3.

### 2.5 Introduction of cloning sites and the DTG mutation into the SCMV genome

To modify SC129f3 for insertion of foreign genes, a set of overlapping PCRs was performed to introduce the multiple cloning site and NIa cleavage site between the P1 and HC‐Pro coding region. PCR A was performed using SC129f3 as template and primer pair VecNotI and 848R+1 (Table S1). PCR B was performed using SC129f3 as template and primer pair 848F+1 and 1028R. The products from A and B were used as template in PCR C with primer pair VecNotI and 1028R. The product from PCR C was digested with *Not*I and *Xho*I and ligated into similarly digested SC129f3 to generate the construct SCMV‐CS1 (cloning site 1). SCMV‐CS2 was made similarly except the primer pair VecNotI and 848R+2 was used in PCR A, and pair 848F+2 and 1028R was used in PCR B. Insertion was confirmed by PCR and sequencing.

To obtain non‐aphid transmissible SCMV clones, a different set of overlapping PCRs was performed to make an alanine to threonine substitution at the sixth amino acid of the coat protein. PCR D was performed using SCMV‐CS1 as template with primer pair 7474F and DAG‐R (Table S1). PCR E was performed using SCMV‐CS1 as template with primer pair DAG‐F and 8510R. The products of D and E were used as template in PCR F with primer pair 7474F and 8510R. The product from PCR F was digested with *Nco*I and *Bsu*36I and ligated into similarly digested SCMV‐CS1 to generate the construct SCMV_DTG_‐CS1.

### 2.6 Insertion of GUS, GFP, and BAR into SCMV vectors

The GUS coding region was amplified using pUGN (Nielsen, Olsen, & Oliver, 1999) as template with primer pair GUSS‐1 and GUSA‐1 or primer pair GUSS‐2 and GUSA‐2 (Table S1). The PCR product was cloned into pGEM‐T Easy to generate pGUS‐1 or pGUS‐2 and verified by sequencing. pGUS‐1 was then digested with *Bsi*WI and *Bgl*II and ligated into similarly digested and dephosphorylated SCMV‐CS1 to generate the construct SCMV‐CS1‐GUS, and pGUS‐2 was digested with *Sac*II and ligated into similarly digested and dephosphorylated SCMV‐CS2 to generate the construct SCMV‐CS2‐GUS. The GFP coding sequence was PCR amplified from pSITE 2CA (Chakrabarty et al., 2007) using primer pair GFPS‐1 and GFPA‐1 or primer pair GFPS‐2 and GFPA‐2 (Table S1). pGFP‐1 and pGFP‐2 were generated by cloning the product into pGEM‐T Easy. After sequence verification, pGFP‐1 was digested with *Bsi*WI and *Bgl*II and ligated into similarly digested and dephosphorylated SCMV‐CS1 to generate the construct SCMV‐CS1‐GFP and pGFP‐2 was digested with *Sac*II and ligated into similarly digested and dephosphorylated SCMV‐CS2 to generate the construct SCMV‐CS2‐GFP. The BAR coding sequence was PCR amplified from pBPMV‐GFP‐BAR (Zhang, Bradshaw, Whitham, & Hill, 2010) as template using primer pair BARS and BARA (Table S1). The product was cloned into pGEM‐T Easy to create pBAR, which was sequence verified. The BAR coding sequence was released from pBAR by *Sma*I digestion and ligated into similarly digested and dephosphorylated SCMV‐CS1 or SCMV‐CS2 to generate the construct SCMV‐CS1‐BAR and SCMV‐CS2‐BAR. For all the constructs, the orientation of the insert was confirmed by PCR and sequencing analysis.

### 2.7 SCMV as an expression vector for GUS, GFP, and BAR

One‐week‐old sweet corn plants were inoculated with the SCMV gene expression constructs by either particle bombardment or mechanical rub‐inoculation. Three weeks later, the colorimetric GUS activity assay was performed on SCMV‐GUS‐infected plants according to (Jefferson, 1987). SCMV‐BAR‐infected sweet corn plants were sprayed with Finale^®^ herbicide, which contains glufosinate–ammonium as the active ingredient (AgrEvo), at a concentration of 0.05% glufosinate–ammonium (w/v) in deionized water. The sweet corn plants were photographed 10 days after herbicide treatment. GFP expression was examined by fluorescence microscopy (Zeiss), and photographs were taken with a digital camera. In all these experiments, non‐infected sweet corn plants and plants infected with the corresponding SCMV empty vectors were included as negative controls.

### 2.8 RNA extraction and RT‐PCR analysis of foreign gene insertions

Leaves of SCMV‐infected or non‐infected plants were harvested for RNA extraction using the RNeasy Plant Mini Kit (Qiagen). After first‐strand cDNA synthesis, primer pairs 745F and 1028R were used to detect the presence of SCMV by RT‐PCR. *Zea mays Actin1* was used as an internal control with primer pair ZmAct1S and ZmAct1R. To test stability of the foreign gene insertions, plants were initially inoculated by particle bombardment. At 3 weeks postinoculation, infected leaves were used as inoculum to rub‐inoculate new plants. Three successive passages were performed. Leaf tissues from the initial inoculated plants and three independent plants in each passage generation were collected for RNA extraction and RT‐PCR analysis.

### 2.9 Enzyme‐linked immunosorbent assay (ELISA) for detecting SCMV

Leaf samples of SCMV‐infected or mock‐treated sweet corn or 10 different inbred line plants were collected for ELISA to detect the infection by SCMV using the ELISA reagent set SRA18100 from Agdia. The assay was performed according to the user guide of the product except a 1‐hr blocking step with 5% non‐fat milk was added between the coating and sample dispensing steps. After adding the PNP substrate, the plate was incubated for 15 min and measured on a plate reader at 405 nm. Grinding buffer only was used as negative control.

### 2.10 Aphid transmission test

Green peach aphids, *Myzus persicae,* maintained on *Brassica juncea* “Tendergreen” were used to test the aphid transmissibility of SCMV containing DAG (wild type) or DTG (non‐aphid transmissible) motif in the viral CP. Aphids were starved for overnight at 4°C in a petri dish and then allowed to feed on maize leaves infected with SCMV_DAG_‐CS1 or SCMV_DTG_‐CS1. After feeding for 2 min, aphids were transferred to non‐infected plants and kept overnight in aphid cages. Insecticide was then sprayed to kill the aphids, and plants were maintained in a growth chamber under normal conditions. Five plants were used for wild‐type and mutated virus, respectively, and ten aphids were used for each plant. The experiment was repeated four times.

## 3 RESULTS

### 3.1 Construction of an SCMV full‐length infectious clone

The SCMV genome was obtained through reverse transcription followed by PCR (RT‐PCR) using total RNA extracted from SCMV‐infected maize tissue. The full‐length genome was placed under control of P35S and Tnos in the same plasmid backbone previously used for a FoMV virus‐induced gene silencing vector (Mei, Zhang, Kernodle, Hill, & Whitham, 2016). Initial screening of SCMV full‐length clones showed that no single clone was infectious when inoculated biolistically onto sweet corn seedlings. However, two pools of clones designated as set 129 (clones SC129, SC159, and SC163) and set 143 (clones SC143, SC147, and SC167) were infectious. The genomes of these six clones were sequenced and compared. Comparison of the predicted viral polyproteins of SC129, SC159, and SC163 identified differences at 15 amino acid positions (Table 1), and SC159 contains a frame shift that leads to early termination of the polyprotein at amino acid 1852. All three clones in set 143 carry the same amino acids at 13 of the 15 positions Q40, I100, P1103, L1216, C1229, M1528, V1536, G1983, L2354, D2504, L2736, F2953, and Q3076, but they differ at positions 555 and 558 (Table 1). With the exception of positions 100, 555, 558, and 2,504, the amino acid residues in the set 143 clones are consistent with the consensus amino acid composition of the 18 full‐length SCMV genomes identified in BLAST sequence alignments when SC129 was used as a query against the GenBank non‐redundant (nr) database (Table 1). Based on these observations, we postulated that Q40, I100, P1103, L1216, C1229, M1528, V1536, G1983, L2354, D2504, L2736, F2953, and Q3076 were the correct amino acid residues at these 13 positions.

**Table 1:** Sequence comparison among SCMV full‐length infectious clones

Clone^a^	SCMV cistron Amino acid position in SCMV polyprotein														
	P1		HC‐Pro		6k1	CI				VPg	NIb			CP	
	40	100	555	558	1,103	1,216	1,229	1,528	1,536	1,983	2,354	2,504	2,736	2,953	3,076
SC159^b^	R	I	S	P	Q	L	R	M	V	‐(G)	‐(L)	‐(D)	‐(L)	‐(F)	‐(Q)
SC163	Q	T	S	P	P	P	C	T	A	E	L	D	P	L	P
SC129	Q	I	F	S	P	L	C	M	V	G	P	G	L	F	Q
Set143^c^	*Q*	I	S/F	P/S	*P*	*L*	*C*	*M*	*V*	*G*	*L*	D	*L*	*F*	*Q*

a Amino acid differences are shown for the three individual clones of Set129 (SC129, SC159, and SC163). For Set143, a summary is provided.

b The predicted amino acid in parentheses is not made due to a frameshift.

c Amino acids in italics are conserved with 18 full‐length SCMV genomes in GenBank nr (release 192).

**Figure 1:**
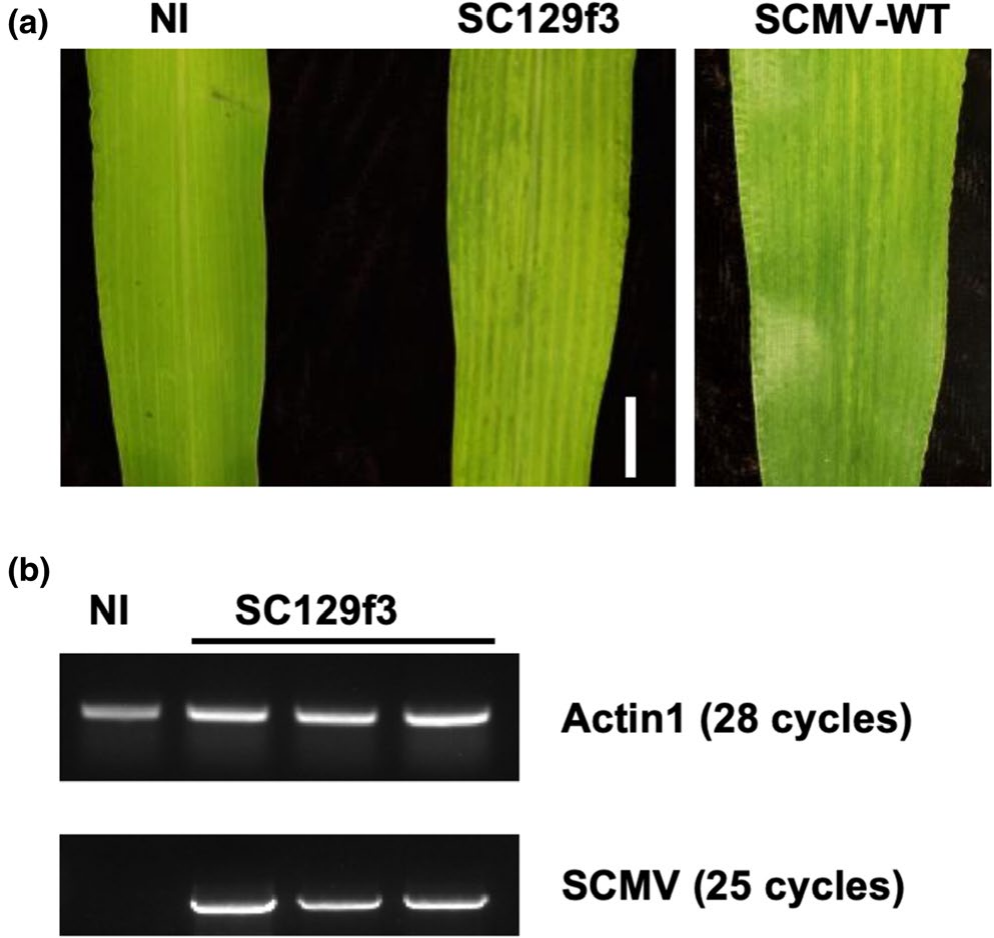
Confirmation of infectivity of the SCMV infectious clone. (a) Typical mosaic symptoms were observed on a SC129f3‐infected leaf but not on non‐infected wild‐type leaf (NI). The mosaic symptoms were indistinguishable from those caused by infection with the wild‐type SCMV (SCMV‐WT). Bar = 1 cm. (b) RT‐PCR amplification using primers for SCMV coat protein sequence on total RNA extracted from a non‐infected plant (NI) and 3 plants inoculated with SC129f3. The SCMV fragment can only be detected in symptomatic leaves of plants inoculated with SC129f3. RT‐PCR amplification of *ZmActin1* was included as an internal positive control for RT‐PCR

We also hypothesized that the preferred amino acids would predominate in virus accumulating in the systemically infected tissues following inoculation with a mixture of the SC129, SC159, and SC163 clones. RT‐PCR was used to amplify five fragments of the viral genome encompassing the 15 amino acid positions. The RT‐PCR products were cloned, and 21 to 36 independent clones of each were sequenced (Table 2). The predominant amino acids at the 15 positions in question were as follows: Q40, I100, S555, P558, P1103, L1216, C1229, M1528, V1536, G1983, L2354, D2504, L2736, F2953, and Q3076 (Table 2). These sequencing results were consistent with our in silico prediction based on sequence comparison of the full‐length SCMV genomes, and they also demonstrated that S at position 555 and P at position 558 are preferred. SC129, which had the fewest differences from the consensus sequence, was modified by introducing amino acid substitutions F555S, S558P, P2354L, and G2504D. The resulting construct was named SC129f3, and it was tested for infectivity following biolistic inoculation of sweet corn plants. Symptoms of leaf mosaic, mottling, and chlorosis occurred in the systemic leaves that were indistinguishable from symptoms caused by the wild‐type virus (Figure 1a). These symptoms were observed as early as 6 days postinoculation (dpi) and persisted in all systemic leaves. RT‐PCR analysis confirmed the presence of SCMV in symptomatic leaves of plants that had been biolistically inoculated with SC129f3 (Figure 1b).

**Table 2:** Predominant amino acids observed in systemically infected plants inoculated with a combination of SC129, SC159, and SC163

Amino acid	Cloned RT‐PCR fragment														
	1		2		3					4			5		
Position	40	100	555	558	1,103	1,216	1,229	1,528	1,536	1,983	2,354	2,504	2,736	2,953	3,076
Residue	Q	I	S	P	P	L	C	M	V	G	L	D	L	F	Q
Number observed	19	20	23	23	25	25	25	32	32	35	32	34	24	22	22
Total clones	21	21	24	24	36	36	36	36	36	36	36	36	24	24	24

### 3.2 Expression of heterologous proteins from modified SCMV clones

**Figure 2:**
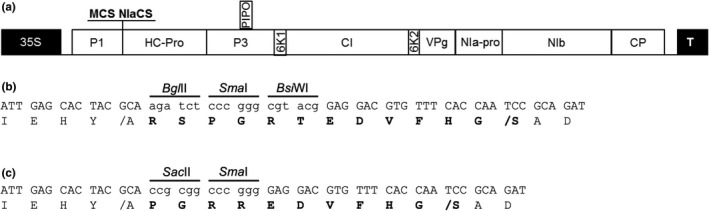
Diagram of SCMV expression constructs and cloning site modifications. (a) Schematic representation of the modified SCMV genome. The positions of the multiple cloning site (MCS) and additional nuclear inclusion a proteinase cleavage site (NIaCS) engineered between P1 and HC‐Pro are indicated. 35S, CaMV 35S promoter; P1, protein 1; HC‐Pro, helper component‐proteinase; P3, protein 3; 6K1, 6 kiloDalton protein 1; CI, cylindrical inclusion; 6K2, 6 kiloDalton protein 2; VPg, genome‐linked viral protein; NIa‐pro, nuclear inclusion a proteinase; NIb, nuclear inclusion b (replicase); CP, coat protein; T, nopaline synthase terminator; and PIPO, pretty interesting potyviral open reading frame. (b) Nucleotide and deduced amino acid sequences of the multiple cloning site in SCMV‐CS1. The *Bgl* II *, Sma*I*, and Bsi* WI sites are shown with lowercase letters, and the P1 and engineered NIa‐Pro cleavage sites are represented by a forward slash. Bold letters indicate amino acids added to create the MCS1 and NIaCS. (c) Nucleotide and deduced amino acid sequences of the multiple cloning site in SCMV‐CS2. The *Sac* II and *Sma*I sites are shown in lowercase letters, and the P1 and engineered NIa‐Pro cleavage sites are represented by a forward slash. Bold letters indicate amino acids added to create the MCS2 and NIaCS

In order to express heterologous proteins from SCMV, two different multiple cloning sites were inserted at the junction of the P1 and HC‐Pro cistrons (Figure 2a). This position has been used successfully for engineering several other potyviral vectors, including SMV (Wang et al., 2006), ZYMV (Arazi et al., 2001), TEV (Dolja et al., 1992), and ClYVV (Masuta et al., 2000). The resulting clones, named SCMV‐CS1 and SCMV‐CS2, harbor different enzyme cloning sites *Bgl*II/*Sma*I/*Bsi*WI and *Sac*II/*Sma*I, respectively (Figure 2b,c). A seven amino acid NIa‐Pro cleavage site derived from the junction of SCMV NIb/CP was introduced after each cloning site (Figure 2b,c). The third nucleotide of each codon was changed to avoid an exact duplication of the RNA sequence encoding the wild‐type NIa cleavage site at the NIb/CP junction. SCMV‐CS1 and SCMV‐CS2 were confirmed to be infectious following biolistic inoculation using the same conditions as for the SC129f3 parental virus clone.

### 3.3 Systemic expression of GFP from SCMV

**Figure 3:**
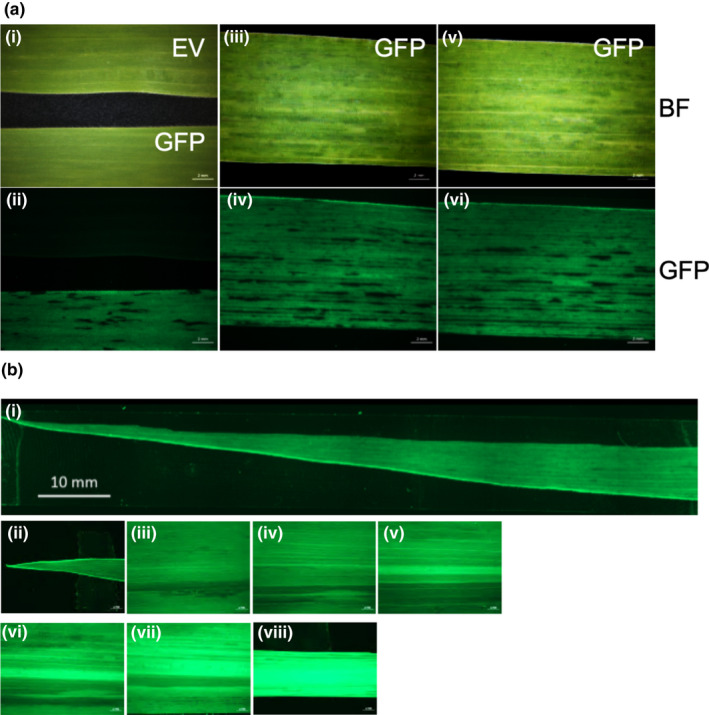
SCMV‐mediated GFP expression in sweet corn (Golden × Bantam). (a) Green fluorescence was observed only in SCMV‐CS1‐GFP‐infected leaves but not in leaves infected with the SCMV‐CS1 empty vector (EV). i, iii, v, bright field; ii, iv, vi, the same leaf as in i, iii, v under green fluorescence channel. (b) i, composite image of green fluorescence of a SCMV‐CS1‐GFP‐infected half leaf (fifth leaf) 10 cm from the leaf tip. ii‐viii, images of a 60 cm SCMV‐CS1‐GFP leaf (fifth leaf) at 10 cm intervals under the green fluorescence channel

To investigate the potential of SCMV for protein expression in maize, the GFP coding sequence minus the stop codon was cloned into SCMV‐CS1 to make pSCMV‐CS1‐GFP. At 2 weeks after inoculation, typical mosaic symptoms were observed on leaves of plants infected with the SCMV‐CS1‐GFP and SCMV‐CS1 empty vector (EV) plants (Figure 3ai,iii,v). The leaves of infected plants were examined using a fluorescent dissecting microscope, and green fluorescence was detected only in SCMV‐CS1‐GFP‐infected leaf tissue (Figure 3aii). The green fluorescence detected in the SCMV‐CS1‐GFP‐infected tissue occurred in a mosaic pattern throughout the leaves. To better visualize the distribution of GFP with respect to mosaic symptoms, we compared bright field and fluorescent images. In general, the lighter green to yellow areas in the bright field image corresponded with green fluorescent signal, whereas the dark green areas in the bright field had relatively less to no green fluorescence (Figure 3aiii‐vi). To examine the expression of GFP across the length of a SCMV‐CS1‐GFP‐infected leaf, a 10‐cm section from the leaf tip was digitally reconstructed from 6 overlapping serial images (2 cm in length for each image) (Figure 3bi). In addition, 7 images (2 cm in length for each image) were taken from a 60‐cm long leaf at 10‐cm intervals (Figure 3bii‐viii). Green fluorescence was seen in all the areas examined, indicating the presence of GFP from the base to the tip of the leaf. GFP was also expressed from the SCMV‐CS2 vector with similar results (Figure S1A).

**Figure 4:**
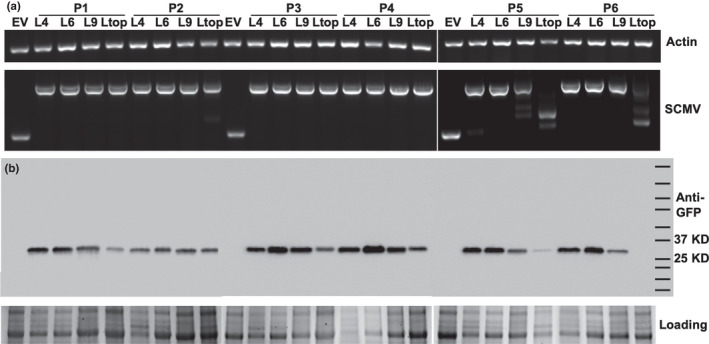
SCMV‐mediated GFP expression in sweet corn (Golden × Bantam). (a) RT‐PCR analyses for the *GFP* insert stability in SCMV‐CS1‐GFP‐infected plants. The upper gel image is the RT‐PCR control showing amplification of a single maize *actin1* mRNA fragment in all samples. The lower gel image is RT‐PCR amplification across the SCMV cloning site. EV indicates the SCMV‐CS1 empty vector that carries no insert. L4, L6, L9, and Ltop indicate the leaf number that was sampled. (b) Western blot analysis showing GFP expression in SCMV‐CS1‐GFP‐infected leaf tissues that are presented in panel A. The upper panel shows the 27 kDa band corresponding to GFP protein detected using anti‐GFP antibody and chemiluminescence. The lower panel shows the protein loading control

To investigate the stability of the *GFP* coding sequence, infected leaves were harvested over a 2‐month time course. RT‐PCR analysis using primers that flanked the GFP insertion site was performed to determine whether the GFP insert was intact or whether deletions occurred. A 344‐nt RT‐PCR product was detected in tissue infected with the SCMV‐CS1 empty vector, and a 1055 nt product was detected in the SCMV‐CS1‐GFP‐infected tissue, indicating the presence of the GFP insert (Figure 4a). A single product of 1055 nt was seen in the 4th and 6th leaf samples. An additional band of smaller size was detected in one of the six leaf 9 samples indicating minor deletion had occurred and increasing numbers of plants were observed with deletions in the top leaf samples. Consistent with the RT‐PCR results, Western blot assay using an anti‐GFP antibody detected GFP in all the L4, L6, and L9 samples and also in most of the top leaf samples where GFP appeared to be less abundant. These results indicate that SCMV‐mediated GFP expression is robust and long‐lasting, but the integrity of the *GFP* insertion may decrease over extended periods of time.

### 3.4 Expression of BAR and GUS from SCMV

**Figure 5:**
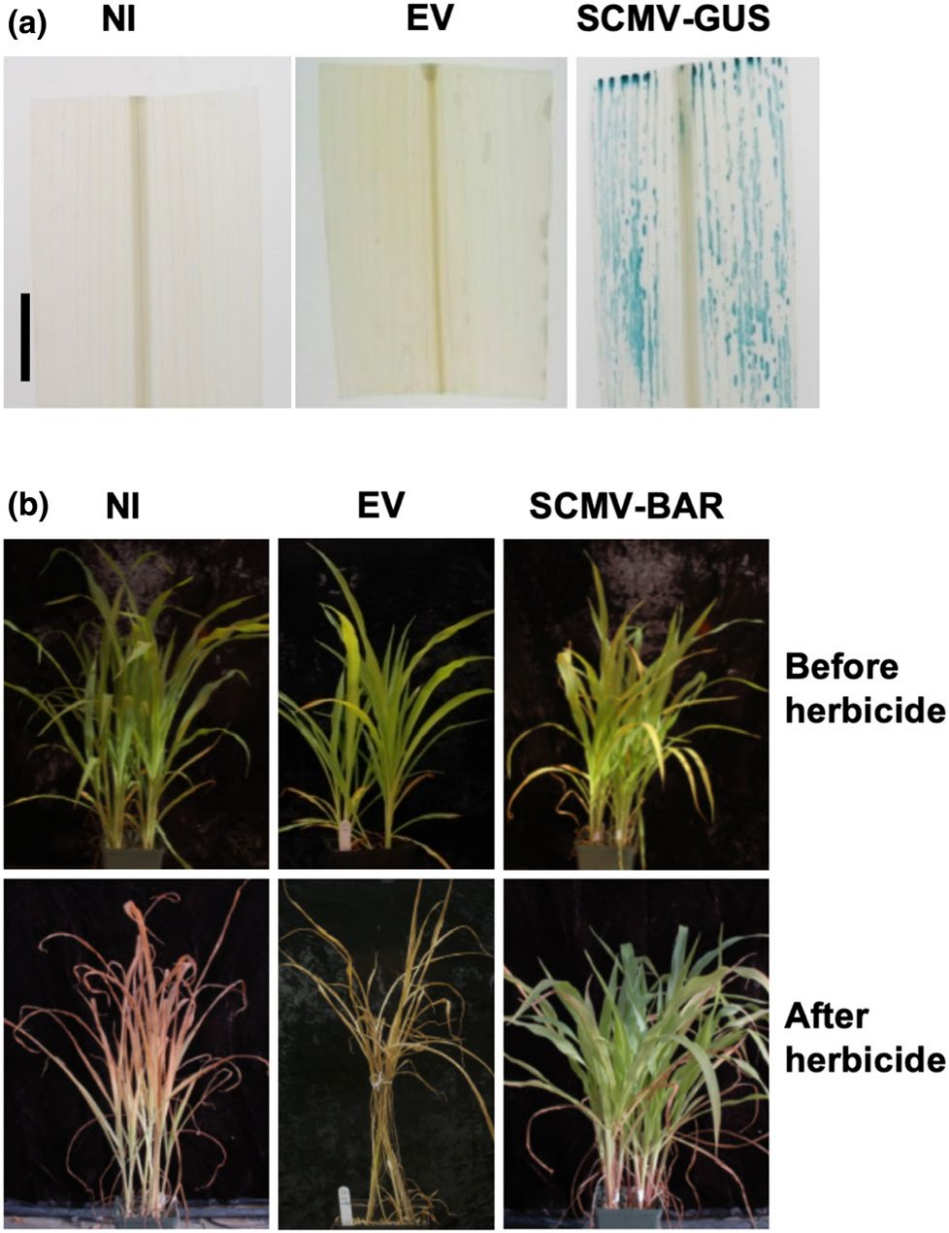
Expression of GUS and BAR proteins from the SCMV expression vector in sweet corn leaves. (a) The leaf on left is from a non‐infected (NI) plant; the middle leaf is from a SCMV empty vector (EV)‐infected plant, and the leaf on the right from a plant infected with SCMV‐CS2‐GUS (biolistically inoculated). Blue indicates the presence of GUS protein in leaves stained with X‐Gluc and cleared with ethanol. (b) SCMV‐CS2‐BAR protects plants from effects of Finale^®^ (Agrevo) herbicide, which contains glufosinate–ammonium as the active ingredient. The herbicide killed non‐infected plants (NI) and plants infected by SCMV empty vector (EV) (Rub‐inoculation R1)

To further investigate the ability of SCMV to express functional proteins of different sizes, the BAR (183 amino acids) and GUS (603 amino acids) proteins were tested. The BAR (549 nucleotides (nt)) or GUS (1809 nt) coding sequences minus the stop codons were cloned into pSCMV‐CS1 or pSCMV‐CS2 to produce pSCMV‐CS2‐BAR or pSCMV‐CS1‐GUS, respectively. Similar to the SCMV clones expressing GFP, the infectivity of SCMV‐CS1‐GUS or SCMV‐CS2‐BAR was confirmed by leaf mosaic symptoms and the expression of functional proteins was then tested. In SCMV‐CS1‐GUS‐infected plants, GUS activity was detected throughout the leaves while no background activity was seen in SCMV‐CS1 empty vector‐infected leaves (Figure 5a). Plants infected by the SCMV‐CS2 empty vector and then sprayed with Finale^®^ (Agrevo) herbicide were killed, whereas all the SCMV‐CS2‐BAR‐infected plants survived (Figure 5b). Similarly, plants infected with the SCMV‐CS1‐BAR virus also survived herbicide application (Figure S2A). These results further confirmed that the modified SCMV vectors have the capability to express different foreign proteins that maintain their biological functions.

### 3.5 Gene expression by SCMV vectors following virus passages

**Figure 6:**
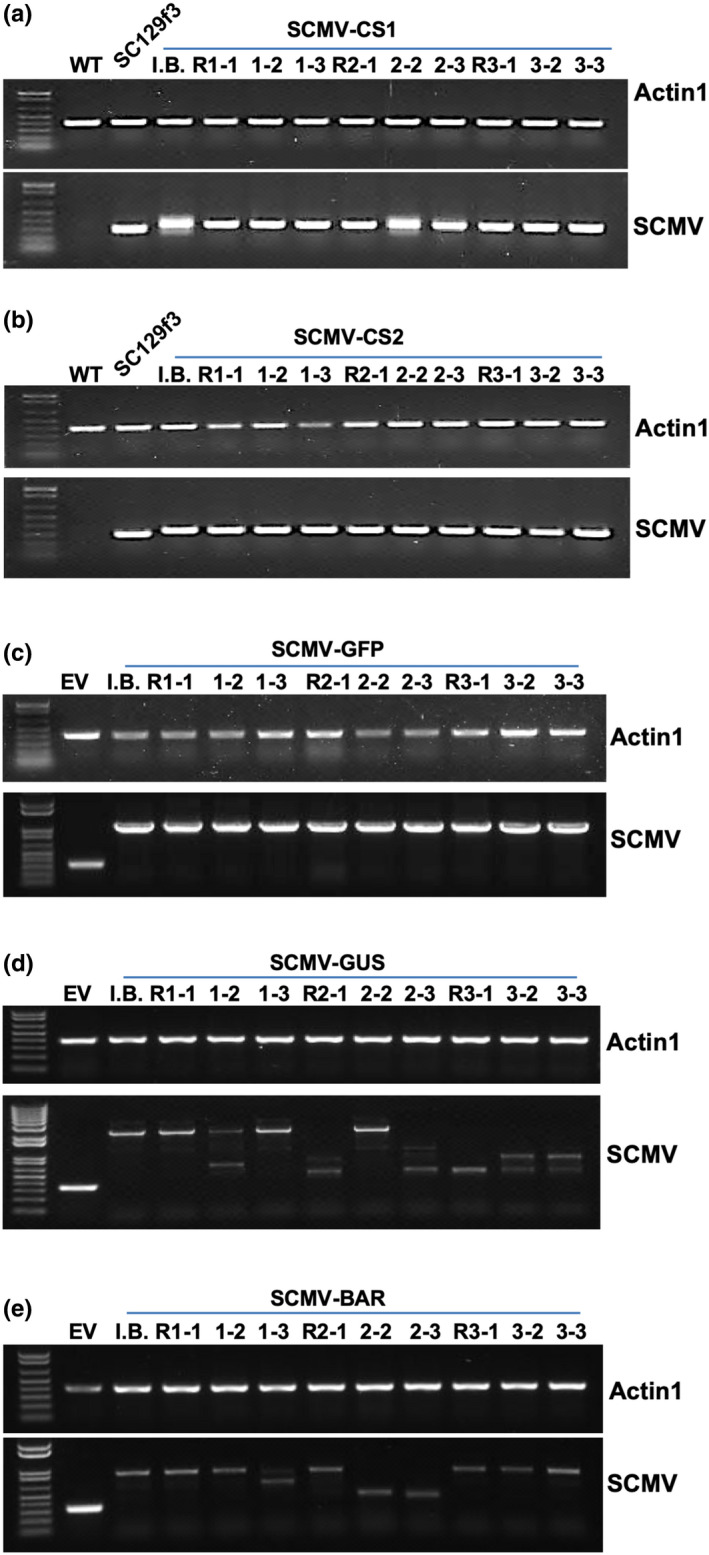
Stability of foreign sequences carried by SCMV vectors. RT‐PCR analysis of plants inoculated with SCMV‐CS1 (a), SCMV‐CS2 (b), SCMV‐CS2‐GFP (c), SCMV‐CS1‐GUS (d), and SCMV‐CS2‐BAR (E). I.B., initial bombardment; R1, rub‐inoculation passage 1; R2, rub‐inoculation passage 2; R3, rub‐inoculation passage 3; EV, empty vector. SCMV primers flanking the insertion site were used to detect the stability of the insertion, and *Zmactin1* was used as internal control

We demonstrated that the SCMV vectors can be successfully used to express three different reporter genes following biolistic inoculation. Next, we tested whether these recombinant viruses could maintain protein expression after they were passed to new plants via rub‐inoculation. To test this, we evaluated the stability of inserted genes following three successive passages by RT‐PCR using primers in the P1 and HC‐Pro cistrons that flanked the cloning site. During each passage, leaves 5 and 6 were collected at 2–3 weeks postinoculation and used as inoculum for the next set of plants and for RNA extraction. As a control, the SCMV‐CS1 and SCMV‐CS2 empty vectors, which have insertions of 42 nt and 36 nt, respectively, were tested first. An unique product of 302 nt was detected in SC129f3‐infected plants indicating infection by the wild‐type SCMV infectious clone while a larger band of approximately 340 nt was detected in all the SCMV‐CS1‐ or SCMV‐CS2‐infected plants demonstrating the stability of the empty CS1 and CS2 modifications (Figures 6a,b).

The stability of GUS, GFP, and BAR was tested in the same way. Only the expected fragment of 1061 nt was detected in the SCMV‐CS1‐GFP‐infected plants (Figure 6c). Furthermore, fluorescence due to the expression of GFP was readily detected in leaf cells from all the plants tested among three passage generations. For GUS, the expected fragment of 2147 nt was detected in the initial biolistically inoculated plant. After serial passages, the 2147 nt band was detected in the first two passage generations along with other smaller bands, indicating partial deletion of the GUS coding sequence (Figure 6d). When GUS activity was tested, 10 of 10 plants from the first passage and 13 of 14 plants from the second passage tested positive. None of the ten plants tested in the third passage possessed GUS activity. The lack of GUS activity after the third passage is consistent with the presence of bands in RT‐PCR assays that were all less than 2147 nt (Figure 6d). When SCMV‐CS2‐BAR‐infected plants were tested, the expected band of 893 nt was detected in the initial biolistically inoculated plant. This band was present in all three of the passage generations although partial deletion was also detected in some plants (Figure 6e). One plant out of eight from the second passage was killed by Finale^®^ (Agrevo) herbicide while all the others survived as a result of expression of the BAR protein (10 of 10 plants survived in serial passage 1, 7 of 8 plants survived in serial passage 2, and 8 of 8 plants survived in serial passage 3) (Figure S2B).

### 3.6 Engineering SCMV to be non‐aphid transmissible

SCMV, like other potyviruses, is naturally transmitted by aphids in a non‐persistent manner (Redinbaugh & Zambrano, 2014). The DAG amino acid motif near the N‐terminus of the CP plays a critical role in the aphid transmissibility of several potyviruses. For example, mutation of DAG to DAL or DAS completely abolished the aphid transmissibility of tobacco vein mottling virus (Atreya, Atreya, & Pirone, 1991; Atreya, Raccah, & Pirone, 1990), and a mutation of DAG to DTG in zucchini yellow mosaic virus rendered the virus non‐aphid transmissible (Gal‐On, Antignus, Rosner, & Raccah, 1992). To prevent aphid transmission of the recombinant SCMV clones, the DAG motif of the SCMV‐CS1 CP was mutated to DTG, and the virulence and aphid transmissibility of SCMV_DAG_‐CS1 and SCMV_DTG_‐CS1 were compared. As expected, SCMV_DAG_ and SCMV_DTG_ caused symptoms that were indistinguishable on sweet corn plants, indicating that the DAG to DTG mutation did not affect SCMV virulence. To test aphid transmission of SCMV_DAG_ and SCMV_DTG_, aphids were allowed to feed on symptomatic plants, and then, 10 aphids were transferred to each of 5 healthy plants and allowed to feed overnight. The aphid‐inoculated plants were grown and examined for symptoms up to 21 dpi. SCMV_DAG_ was transmitted by *M. persicae* in the range of 40%–100% with a mean of 65%. In four replications of the experiment, 2 of 5, 2 of 5, 4 of 5, and 5 of 5 plants developed symptoms. However, 0 of 5 plants infected with SCMV_DTG_ were symptomatic in each of the four replications of the experiment. These data demonstrate that SCMV clones carrying the DTG mutation in the CP cannot be transmitted by *M. persicae*.

### 3.7 SCMV infection of maize inbred lines

To test the potential of the SCMV expression system to be used in different maize genetic backgrounds, seedlings of 10 different inbred lines of dent corn were rub‐inoculated with the SCMV wild‐type parental virus. Mosaic symptoms were observed on leaves of all the maize inbred lines tested, including B73, Mo17, Mo47, B101, B104, W22CC, K55, FR1064, A188, and W64A (Figure S3A). An ELISA test was performed to confirm SCMV infection in the systemic leaves of the 10 inbred lines (Figure S3B). We rub‐inoculated B73 seedlings with SCMV‐CS1‐GFP and observed GFP expression similar to that in sweet corn demonstrating the potential for protein expression in dent corn inbred lines (Figure S1B). These results indicate the SCMV expression vectors can be used in a wide variety of genetic backgrounds of interest to the maize research community.

## 4 DISCUSSION

We report the development of a full‐length SCMV infectious clone and its modifications for gene expression in maize. The junction of the P1 and HC‐Pro cistrons was engineered to include a cloning site for inserting coding sequences of interest followed by a NIa protease cleavage site. This cloning strategy requires the proteins of interest to be expressed in‐frame with the viral polyprotein and then processed into their free forms by the viral‐encoded P1 and NIa proteases. Two versions of the multiple cloning site (CS1 and CS2) were made to provide different choices of restriction enzyme sites as dictated by the nucleotide sequences encoding proteins of interest. Both versions of the cloning site were confirmed to be stable after three serial passages in sweet corn using the marker gene *GFP* and the herbicide resistance gene *BAR*. The possibility and efficiency of protein expression using other regions such as the NIb/CP junction, which have also been shown to be useful in potyviruses (Kelloniemi et al., 2008), remain to be further explored in the future. By mutating the DAG motif in the SCMV‐CP into the non‐aphid transmissible DTG version, we generated a non‐aphid transmissible variant that prevents insect‐vectored transmission of the recombinant virus.

We demonstrated that three reporter proteins, GUS (1.8 kb), GFP (0.72 kb), and BAR (0.55 kb), were functional in sweet corn plants when expressed from SCMV. SCMV‐mediated GFP expression was observed throughout leaves in a mosaic pattern that coincided with viral symptoms. When inoculated at the two leaf stage, GFP expression was readily detected in L4 up to the top leaf, which was just below the tassel. The number of the top leaf varied but was usually L12–L14. The ability of SCMV‐GFP to express GFP over a developmental time course and following three serial passages demonstrates that the GFP coding sequence is relatively stable. SCMV‐mediated GUS expression was also observed in a mosaic pattern throughout the infected leaves and was detected from L4 to L11. The pattern of GUS expression appeared to be less uniform than GFP, most likely because it was not possible to uniformly infiltrate the maize leaves with the solution containing the X‐Gluc substrate. SCMV‐GUS could only be passaged twice before GUS coding sequence was lost due to deletions indicating that it is less stable than GFP and BAR. The stability of GFP and BAR inserts relative to GUS implies that larger coding sequences have a greater probability to become deleted after serial passages. Because systemic GUS activity was detected in the primary inoculated plants and the first two passage generations, we conclude that the SCMV genome can tolerate an insertion size of at least 1809 nt, but we do not know the upper limit. Although GUS expression has been reported in several potyvirus vectors, the stability of GUS is poor in some cases (Arazi et al., 2001; Beauchemin, Bougie, & Laliberte, 2005). The relatively stable expression of GUS demonstrates that SCMV is suitable for analyzing the functions of proteins of at least 600 amino acids.

This work adds SCMV to a growing list of viral vectors that are capable of systemic gene expression in cereals, each with their inherent advantages and limitations. Barley stripe mosaic virus (BSMV) is widely used for gene silencing and expression in barley and wheat (Lee, Hammond‐Kosack, & Kanyuka, 2012, 2015). BSMV is known to infect maize, but it has only recently been demonstrated to have potential use as a gene silencing or gene expression vector in maize (Cheuk & Houde, 2018; Jarugula, Willie, & Stewart, 2018). An improved BSMV expression vector was recently reported that enables the co‐expression of two proteins and increased capacity of the virus to express a coding sequence of up to 2.1 kb (Cheuk & Houde, 2018). Using this vector, the authors reported GFP expression in the shoots and roots of *Zea mays* var. *everta* (popcorn) seedlings. Wheat streak mosaic virus (WSMV), a *Tritimovirus* similar to potyviruses, has been reported for protein expression in wheat and maize (Choi, Stenger, Morris, & French, 2000; Tatineni, McMechan, Hein, & French, 2011). Like SCMV, foreign proteins must be expressed initially as fusions to the WSMV polyprotein. Proteolytic cleavage sites are introduced in the viral genome to release the expressed proteins through the activity of viral‐encoded proteinases. In the case of WSMV‐mediated GFP expression in maize, aggregate‐like fluorescent bodies were observed because of incomplete cleavage (Tatineni et al., 2011). The cleavage of foreign proteins in the SCMV expression system appears to be highly efficient, because there was no aggregation of GFP fluorescence and immunoblot analysis detected GFP only at the size of its 27 kDa free form (Figure 4, Figure S1).

Bouton et al. (2018) recently reported a vector based on FoMV that can be used for heterologous protein expression in wheat and maize. FoMV uses a subgenomic mRNA strategy to express its genes that are downstream of the viral replicase, which is in contrast to the polyprotein strategy utilized by SCMV. In FoMV, a 101 nt sequence spanning the CP promoter region was duplicated and placed up stream of the wild‐type CP promoter. This duplicated promoter sequence was followed by a multiple restriction enzyme cloning site for insertion of genes of interest. This FoMV was shown to express GFP and GUSPlus in wheat and maize, as well as the necrotrophic fungal effector (ToxA) in wheat. The FoMV vector was created in a binary plasmid backbone, and it could be agroinoculated into *Nicotiana benthamiana* leaves followed by rub‐inoculation onto leaves 1, 2, and 3 of wheat or maize seedlings. The virus could express GFP and GUSPlus in the inoculated leaves and in the systemic leaves. GFP fluorescence was reported in systemic leaves of maize plants for greater than 2 weeks and to at least leaf 7. It appears that GFP fluorescence was not monitored after plants had developed 7 leaves. The expression of GUSPlus was also observed in the systemic leaves of maize plants, but the pattern of expression suggested a peak of expression in leaf 5 with patchy GUSPlus activity reported in leaf 6. This pattern of expression combined with the observation that 33%–83% of symptomatic plants expressed GUSPlus activity suggests that the 1.8 kb GUSPlus insert is much less stable in FoMV than the 0.7 kb GFP insert. Although no molecular analyses were presented to enable direct assessment of the frequency and extent of deletions of foreign sequences from FoMV, a comparison to our phenotypic data suggests that foreign inserts are more stable in SCMV than in the FoMV expression vector in the initially inoculated plants. However, the FoMV system has advantages such as the generation of inoculum by the simple and efficient procedure of agroinoculation into *N. benthamiana*, and there is no requirement for proteins to be expressed in‐frame with the viral polyprotein. *N. benthamiana* is not a host for SCMV and, thus, cannot be used for agroinoculation of SCMV infectious clones.

Maize is an important model for genetics and plant biology, and in addition, it is an important grain crop that is widely cultivated throughout the world. It is used in livestock feed and processed into a multitude of food and industrial products including starch, sweeteners, corn oil, beverage and industrial alcohol, and fuel ethanol. The current analysis of the maize B73 reference genome (B73 RefGen_v4) predicts 39,498 coding and 6,774 non‐coding genes (gramene.org, accessed 10/8/2018) (Schnable et al., 2009). Analysis of the function of these genes could be facilitated by new tools, such as viral vectors, that enable rapid analysis of gene functions through VIGS or protein expression. We expect that the SCMV vectors described herein represent a useful addition to the toolkit used for evaluating the functions of genes in maize. We confirmed that our SCMV isolate is able to infect at least ten different inbred lines of dent corn, as well as sweet corn, suggesting that it may be useful in a broad range of important maize genotypes. In addition, the potential host range of SCMV includes other agriculturally important monocot crop plants such as sorghum, sugarcane, rice, rye grass, barley, and miscanthus (Pirone, 1972). Thus, this SCMV vector may also prove useful for research on many other economically important plant species. Another important area of future investigation is to explore the potential to use SCMV to simultaneously express multiple proteins as has been shown for other potyviruses (e.g., Kelloniemi et al., 2008; Seo, Choi, & Kim, 2016), and recently, in maize from the cytorhabdovirus barley yellow striate mosaic virus (BYSMV) (Gao et al., 2019). Finally, SCMV is an important pathogen by itself or in co‐infections with other viruses (Redinbaugh & Stewart, 2018; Redinbaugh & Zambrano, 2014; Wu, Zu, Wang, & Chen, 2012). The development of the infectious clone and expression of reporter proteins is expected to provide valuable resources for better understanding the biology of SCMV and its interactions with other viruses and its hosts.

## CONFLICT OF INTEREST

The authors declare no conflict of interest associated with the work described in this manuscript.

## AUTHOR CONTRIBUTIONS

Y.M., C.Z., J.H.H., and S.A.W. conceived and designed the research plan; Y.M., G.L. performed the experiments; Y.M., G.L., C.Z., J.H.H., and S.A.W. analyzed the data; Y.M. and S.A.W. wrote the manuscript with contributions from all authors. S.A.W. agrees to serve as the author responsible for contact and ensures communication.
