# Supplementary figures and images for: A sugarcane mosaic virus vector for gene expression in maize
Source: Plant Direct. 2019 Aug 8;3(8):e00158. doi: 10.1002/pld3.158 (PMC6686331; doi:10.1002/pld3.158)

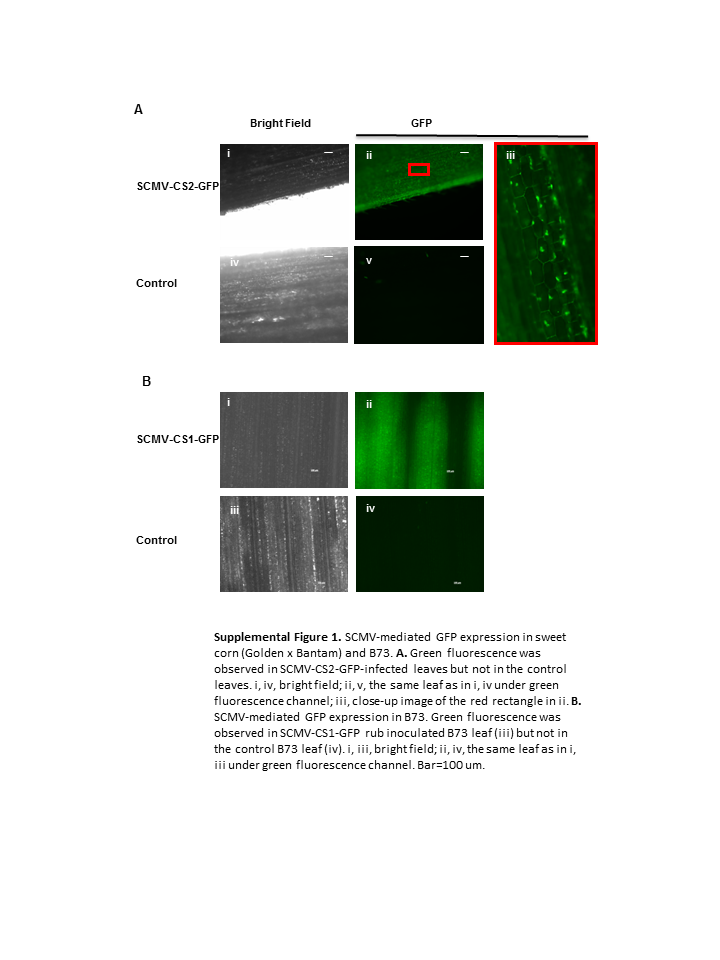

Supplement: Supplementary file 1 [file PLD3-3-e00158-s004.TIF]

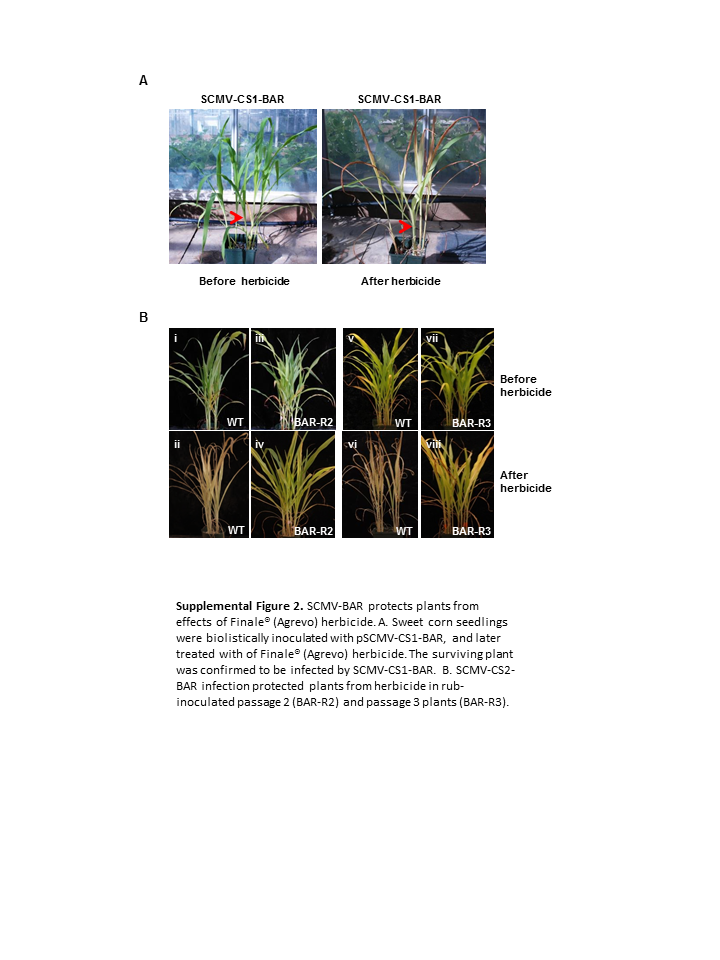

Supplement: Supplementary file 2 [file PLD3-3-e00158-s003.TIF]

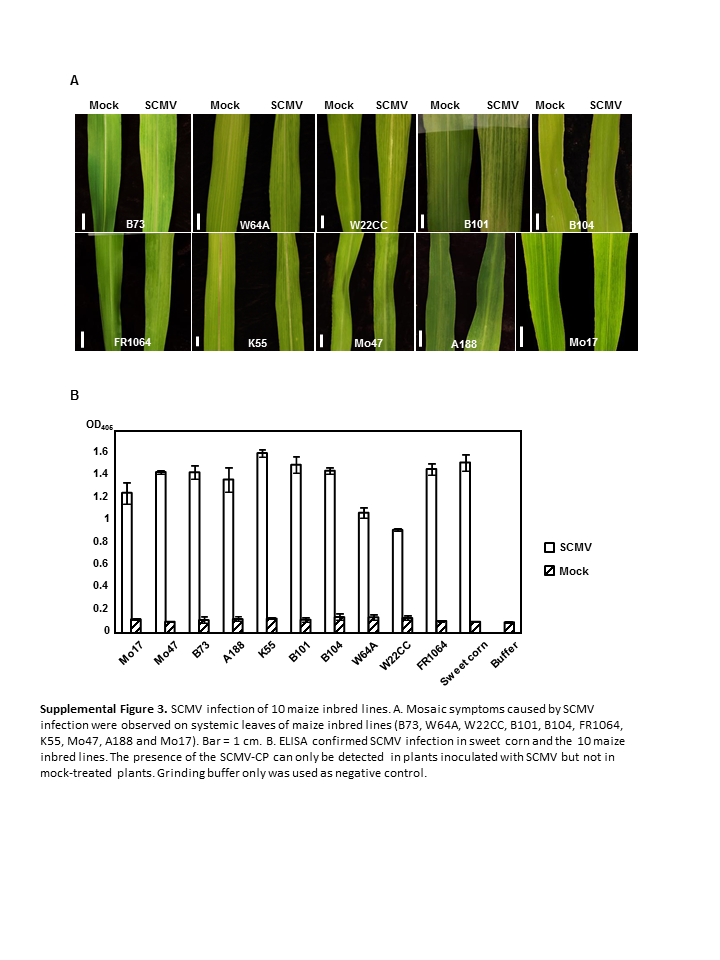

Supplement: Supplementary file 3 [file PLD3-3-e00158-s005.TIF]
