# Supplementary material for: A sugarcane mosaic virus vector for gene expression in maize
Source: Plant Direct. 2019 Aug 8;3(8):e00158. doi: 10.1002/pld3.158 (PMC6686331; doi:10.1002/pld3.158)
Supplement: Supplementary file 4 [file PLD3-3-e00158-s002.docx]

**Supplemental Table 1.** Oligonucleotide primers used in this study.

| **Name** | **Sequence (5’ -> 3’)** |
| --- | --- |
| ***Primers used to clone and sequence full-length SCMV genomes*** | |
| SC-5end | AAAAACAACAAAACTCAACACAACACAACAAAA |
| SC-415R | TGCTTTGTTCGCGGATTTTCCA |
| SC-745F | GAGGGAGCAGTGGTCTCA |
| SC-2120R | CGCGCATTTCACTATCCATAGA |
| SC-2859F | ACAATATATCAGCTACGCATCTAA |
| SC-2859R | TTAGATGCGTAGCTGATATATTGT |
| SC-2916F | AGCTCTACTTTAACCAAACTCCG |
| SC-2916R | CGGAGTTTGGTTAAAGTAGAGCT |
| SC-3239R | TGACACCTGTGTGAGTTAAGT |
| SC-3601F | TGAAGATTGGTGGTCAAATCAA |
| SC-3748F | TAGAGGAGCAGTCGGCTCGGGAA |
| SC-3748R | TTCCCGAGCCGACTGCTCCTCTA |
| SC-4404F | GAGAATGGCGTCACACTAGA |
| SC-4533F | CGATTGGGTCGTGTTGGCA |
| SC-5244F | ATGAAAGATCACACGAAGGA |
| SC-5350F | GGCTCTCAACACAGTTATTCA |
| SC-5647F | TCGGATCTGCTTACACTAAGAA |
| SC-6004F | TAGCAGGTTTCCCAGAGTATGA |
| SC-6495F | AGTGTAACAGCACCAAAAGGAA |
| SC-6721F | ACAAGTGGGAAAAAGGATGGCA |
| SC-6721R | TGCCATCCTTTTTCCCACTTGT |
| SC-7014F | TATGACAAAAGCAGATTAAACAGA |
| SC-7788F | GTCGACAACACACTCATGGT |
| SC-8200F | GGCAACTTGGCGTATGGAA |
| SC-8690F | GAAAATGCGCTTACCTAAAGCAA |
| SC-8870R | GTGTCATCAATTTCGTATTCCT |
| SC-9118F | CAATCTCACCGACTATAGCTTA |
| SC-9200R | TGGCATCATACCATCTATCAAACT |
| SC-3end | TTTTTTTTTTTTTTTTTTTTGTCTCTCACCAAGAGACTCGCA |
| 35S-Seq | ACG CAC AAT CCC ACT ATC |
| Nos-Rev | AGA CCG GCA ACA GGA TTC A |
| ***Primers used to amplify the five fragments encompassing the 15 amino acid differences*** | |
| 157F | GAACGTGGACCTACGTGACA |
| 745R | GTGAGACCACTGCTCCCTCT |
| 1487F | TAGGGAATACCACGCCAAAC |
| 2120R | CGCGCATTTCACTATCCATAGA |
| 3338F | TGATCCACAGAAAAGCGATG |
| 4955R | CGGAATTTTGACGTGGTCTT |
| 6015F | CCAGAATATGAAGGAACACTTC |
| 7897R | TCATCACCATTCGCAAACAT |
| 8232F | GCAAATCTCGCAAAAGAAGG |
| 9614R | CCAAGAGACTCGCAACACAA |
| ***Primers used to introduce cloning sites and the NIa-Pro cleavage site*** | |
| VecNotI | AAGGAGCTGACTGGGTTGAA |
| 848R+1 | ACACGTCCTCCGTACGCCCGGGAGATCTTGCGTAGTGCTCAATATCCAA |
| 848F+1 | CGGGCGTACGGAGGACGTGTTTCACCAATCCGCAGATCCCCAGGCTAAC |
| 848R+2 | AAACACGTCCTCCCCGGGCCGCGGTGCGTAGTGCTCAATATCCAA |
| 848F+2 | CCCGGGGAGGACGTGTTTCACCAATCCGCAGATCCCCAGGCTAAC |
| 1028R | GCATGTCTTGCATGTAATTTTGA |
| ***Primers used to introduce the DTG mutation in CP*** | |
| 7474F | TTCACATCATTTAGAAGGTCCA |
| DAGR | CCTTGTGCACCCGTATCAA |
| DAGF | TTGATACGGGTGCACAAGG |
| 8510R | TACACCAGTTCCAGCTCCTG |
| ***Primers used to insert GUS, GFP, and BAR coding sequences in SCMV-CS1 and SCMV-CS2*** | |
| GUSS-1 | GAAGATCTATGGTCCGTCCTGTAGAAACC |
| GUSA-1 | CCGCGTACGTTGTTTGCCTCCCTGCTG |
| GUSS-2 | TCCCCGCGGATGGTCCGTCCTGTAGAAACC |
| GUSA-2 | TCCCCGCGGTTGTTTGCCTCCCTGCTG |
| GFPS-1 | GAAGATCTATGGTGAGCAAGGGAGAGGA |
| GFPA-1 | CCGCGTACGCTTGTACAGCTCGTCCATGC |
| GFPS-2 | TCCCCGCGGATGGTGAGCAAGGGAGAGGA |
| GFPA-2 | TCCCCGCGGCTTGTACAGCTCGTCCATGC |
| BARS | TCCCCCGGGATGAGCCCAGAACGACG |
| BARA | TCCCCCGGGGATCTCGGTGACGGGCA |
| ***Primers used to amplify maize actin gene (control for RT-PCR)*** | |
| ZmAct1F | CCTGAAGATCACCCTGTGCT |
| ZmAct1R | GCAGTCTCCAGCTCCTGTTC |
