# Supplementary material for: A sugarcane mosaic virus vector for gene expression in maize
Source: Plant Direct. 2019 Aug 8;3(8):e00158. doi: 10.1002/pld3.158 (PMC6686331; doi:10.1002/pld3.158)
Supplement: Supplementary file 5 [file PLD3-3-e00158-s001.pdf]

Reviewer #1 (Comments for the Author):

The manuscript aims to develop a new viral overexpression system for maize.
Major points
1) Introduction: The introduction states that no viral vector for efficient in planta heterologous gene expression has been broadly adopted and utilized by the maize research community for functional genomics... This statement is so narrow that it could apply to almost any viral expression system with any plant species. In fact many viruses are able to infect maize and other cereals (see Table I in DOI: 10.3923/ijv.2010.126.137). In particular, the BSMV and the WSMV were previously used to express proteins in maize. This is acknowledged in the discussion with skewed excuses to indicate that they may not be appropriate (until proven to the contrary). This makes a rather weak excuse to develop a new system for gene expression in maize. However, if it was a very efficient system, it could find its place among maize researchers. Unfortunately, despite the work done, this is not the case.

2) Results :
- There is a fairly lengthy process to obtain a functional SCMV infecting strain. I will develop more on this aspect in the minor comments.

- The expression of the reporter gene GFP is very patchy and uneven. (Fig. 3A iv and vi). This is quite different than expression using transgenic plants since it results in many cells that do not express the foreign gene. The expression of the GUS reporter gene is even more patchy (Fig. 5A). This could explain in part the effect of the bar gene which does not completely protect maize plants against herbicide treatment (Supplementary Fig. 2).

3) Discussion:
- The claim is that the SCMV system can tolerate insertion size up to 1809 nt is not justified since GUS expression is very patchy and both BAR and GUS are fairly unstable with deletions observed after the first generation (Fig. 6D and E).

- The WSMV was reported in the discussion and claimed to be limited to the specific maize inbred line SDp2. This statement is wrong since nothing indicates a limitation about the number of maize lines in Choi et al. (2000). Tatineni et al. actually mentioned this strain to indicate that GUS expression may limit the host range but GFP expression worked fine in SDp2.

- The discussion indicates that An improved BSMV expression vector was recently reported that enables the co-expression of two proteins and increased capacity of the virus to express a coding sequence of up to 2.1 kb (Cheuk and Houde 2018). Although BSMV is known to infect maize, it has only recently been demonstrated to have potential use as a vector, at least for VIGS, in some maize inbred lines (Jarugula et al. 2018). There is an important information missing from the first paper (Cheuk and Houde 2018). The overexpression of GFP was actually shown to function in maize (see Supplementary Figure 2 of this paper).

Minor points :
- Three different SCMV lines (SC129, SC159 and SC163) are coexpressed to select the best strain.

However, the SC159 strain is indicated to have a frameshift mutation. It should not have been used for selection since it would not likely process the polyprotein properly. The analysis in Table 2 thus loses much of its logic and simply looking at the mosaic symptoms with the different lines should have been sufficient. If the objective was to have s sequence corresponding to the Genbank sequence, this could have been simply stated and this would have bee clear and sufficient since the it appears on the end that the objective is to correspond to the Genbank sequence.

- Gene expression in different inbred lines is shown in Supplemental Fig. 3. It is very difficult to see the expression in these images. Since a GFP construct is available, GFP images should be shown. This is much more informative than an ELISA assay on whole leaves.

- In Fig. 1A, it is difficult to understand the regions being recombined. All the restriction site positions should be indicated on the different sequence representation. In Fig. 1C, the 30 cycle of PCR does not add anything and should be removed.

- Several references are missing : Xia et al. 1999; Nielson et al. 1999; Chakrabarty et al. 2007; Zhang et al. 2010

I will not take the time to go through very minor revisions. Overall, despite some interesting findings, this manuscript does not provide a system that allows a stable and uniform expression of large proteins in maize.

Reviewer #2 (Comments for the Author):

The manuscript by Mei et al. describes development of a new virus-based vector for protein expression in maize. This is a simple and straightforward study whereby a Sugarcane mosaic potyvirus (SCMV) has been converted into a vector using a now classic strategy of engineering a multiple cloning site at the junction of the P1 and HC-Pro cistrons for insertion of foreign sequences. This strategy clearly also worked well for SCMV, and the authors were able to express GFP, BAR and GUS proteins in systemically infected maize plants. While expression of GFP was stable in leaves of maize plants of different age, the larger genes (in particular GUS, which is fairly long ~ 1.8 kb) were less stable in the SCMV vector, which is expected based on the studies from other potyvirus based expression systems. Interestingly, GFP expression was also found to be stable when the recombinant SCMV was passed to new plants via rub-inoculation for up to 3 subsequent rounds. But again, BAR and especially GUS gene containing vectors were a lot less stable. Nevertheless, even after a second passage SCMV expressed detectable GUS activity in most of the inoculated plants, and most of the plants infected with SCMV:BAR survived treatment with herbicide (Finale). The authors then demonstrated that their new SCMV vectors could be used for protein expression in at least 10 different maize genotypes representing different genetic backgrounds. Finally, the SCMV vector has been further modified/mutated to ensure the engineered virus can no longer be transmitted by aphids, which makes the new vector more safe to use in the laboratory.

Overall, the manuscript is very clearly written and well illustrated. This new SCMV-based vector for systemic protein expression in maze will of interest to the maize research community alongside other vectors such as BSMV, WSMV and FoMV. If anything, one potential disadvantage of this vector is that it needs to be delivered to maize leaves using microprojectile bombardment, which requires a fairly expensive specialist equipment.
